# VirulentPred: a SVM based prediction method for virulent proteins in bacterial pathogens

**DOI:** 10.1186/1471-2105-9-62

**Published:** 2008-01-28

**Authors:** Aarti Garg, Dinesh Gupta

**Affiliations:** 1 Structural and Computational Biology Group, International Centre for Genetic Engineering and Biotechnology (ICGEB), Aruna Asaf Ali Marg, New Delhi 110067, India

## Abstract

**Background:**

Prediction of bacterial virulent protein sequences has implications for identification and characterization of novel virulence-associated factors, finding novel drug/vaccine targets against proteins indispensable to pathogenicity, and understanding the complex virulence mechanism in pathogens.

**Results:**

In the present study we propose a bacterial virulent protein prediction method based on bi-layer cascade Support Vector Machine (SVM). The first layer SVM classifiers were trained and optimized with different individual protein sequence features like amino acid composition, dipeptide composition (occurrences of the possible pairs of i^th ^and *i*+1^th ^amino acid residues), higher order dipeptide composition (pairs of i^th ^and *i*+2^nd ^residues) and Position Specific Iterated BLAST (PSI-BLAST) generated Position Specific Scoring Matrices (PSSM). In addition, a similarity-search based module was also developed using a dataset of virulent and non-virulent proteins as BLAST database. A five-fold cross-validation technique was used for the evaluation of various prediction strategies in this study. The results from the first layer (SVM scores and PSI-BLAST result) were cascaded to the second layer SVM classifier to train and generate the final classifier. The cascade SVM classifier was able to accomplish an accuracy of 81.8%, covering 86% area in the Receiver Operator Characteristic (ROC) plot, better than that of either of the layer one SVM classifiers based on single or multiple sequence features.

**Conclusion:**

VirulentPred is a SVM based method to predict bacterial virulent proteins sequences, which can be used to screen virulent proteins in proteomes. Together with experimentally verified virulent proteins, several putative, non annotated and hypothetical protein sequences have been predicted to be high scoring virulent proteins by the prediction method. VirulentPred is available as a freely accessible World Wide Web server – VirulentPred, at http://bioinfo.icgeb.res.in/virulent/.

## Background

Virulence of a bacterial pathogen is its relative ability to cause a disease usually described in terms of number of infecting bacteria, the route of its entry into the host body and intrinsic bacterial virulence factors. The bacterial virulence factors are commonly virulent proteins, carbohydrates and other molecules synthesized by bacterial enzymes. The intrinsic virulence factors are under selective pressure which is needed for the bacteria to survive and proliferate in the host cells despite the high mutation rate in bacterial species. The clinical manifestation of a disease depends on the interaction of virulent factors with the host cells and immune system. The most important virulent factors – the virulent proteins are coded in the genes present in the chromosomal DNA or mobile genetic elements like bacteriophages or plasmids [1,2]. Virulent proteins have been further classified on the basis of mechanisms of virulence. Adhesins belong to an important class of bacterial proteins, which play an important role in the process of adherence of bacteria to the host cells. This class of proteins includes fimbria and pili found in *Escherichia coli*, *Vibrio cholerae*, *Pseudomonas aeruginosa *and *Neisseria *species. Adhesins are important vaccine candidates as these bacterial proteins are surface exposed. Colonization factors are a class of proteins, which enables certain bacteria to colonize within the host cells, for example *Helicobacter pylori *survives in the acidic milieu of the human stomach by producing urease enzyme, which catalyzes the formation of carbon dioxide and ammonia that can neutralize the acidic pH. The virulence of different strains of *Helicobacter pylori *correlates with the level of production of urease. Certain bacteria produce a class of proteins called invasion factors, which disrupt the host cell membranes and stimulates endocytosis, facilitating the entry of bacteria into the host body across protective epithelial tissue layers. Similarly, few bacteria are known to produce proteins that bind to the host antibodies. The most commonly known virulence factors are the bacterial toxins that poison the host cells and cause tissue damage. In addition, other elements such as cell surface carbohydrates and proteins that protect pathogens from host defense mechanisms are included in the class of defensive virulence factors which includes capsular polysaccharides, lipopolysaccharides and outer membrane proteins. Apart from these, there are other virulence traits which are indirectly involved in virulence, such as secretory machineries, siderophores, catalases and regulators; which are equally essential for pathogens to manifest infection [3].

Microbial pathogens are responsible for the most devastating diseases and widespread epidemics. However, with the advancements in medical research and availability of effective antimicrobial regimens, the disease burden has reduced remarkably. However, the threat due to certain pathogens is rising due to drug resistant strains and emergence/reemergence of infectious agents, which poses a cause for alarm [4]. The microbial genome sequencing projects have given more insight into microbial pathogenesis and drug resistance, opening new avenues for microbial research. With the completion of first pathogen genome sequencing (*Haemophilus influenzae *genome) in 1995 [5], the number of accomplished sequencing projects has been increasing exponentially. According to a recent report, more than 532 microbial genomes have been sequenced, and many more genomes are expected to be sequenced in the next few years [6]. The complete sequences of pathogen genomes have provided wealth of information about the determinants of bacterial virulence, however due to diversity and complexity of virulence proteins, the computational tools for interpretation; identification and characterization of virulence-associated proteins are still limited. Moreover, a large number of predicted proteins in the microbial genomes are yet to be assigned any function, it is beyond doubt that many of these are virulence associated proteins. Hence, availability of prediction methods for virulent proteins will enhance knowledge about bacterial virulence, annotations of (novel) virulent genes and development of novel antimicrobial targets. Similarity search methods like BLAST [7] are expected to distinguish between virulent and non-virulent proteins with reasonable accuracy. However, the choice of this method may not be reasonable in the cases where virulent proteins are evolutionarily distant and do not have significant sequence similarity to known virulent protein sequences. Several computational strategies have been proposed to deal with the problems of finding sequences with remote similarity and homology. PSI-BLAST is one such algorithm, which aids in identification of remotely similar proteins [8]. Another reasonable method to overcome this limitation is the machine learning algorithms. Recently, a publication by Sachdeva *et al*., [9], details a neural network based prediction of virulence factors with a sensitivity of 89%; albeit specific only for adhesins. In this work we have developed a Support Vector Machine (SVM, [10]) based method for prediction of virulent protein sequences. Different SVMs classifiers were trained with sequence features of bacterial virulent proteins such as amino acid compositions (AAC), dipeptide (i^th ^and i+1^th ^amino acids pairs), higher order dipeptide (i^th ^and i+2^nd ^amino acid pairs) composition, evolutionary information in the form of PSSM profiles, based on PSI-BLAST similarity search and combinations of the above mentioned features. Finally, we developed a bilayer cascade SVM in which the results from the first layer (SVM scores from SVMs based on AAC, dipeptide, higher order dipeptide composition and PSI-BLAST results) were cascaded to train and generate the second layer final SVM classifier. The bilayer cascade SVM turned out to be the most efficient in differentiating virulent proteins from non virulent ones. A genera-wise breakup of virulent protein sequences used for SVM training in the current study is given in the Table 1.

**Table 1:** Genera of bacterial species and corresponding number of virulent protein sequences used for positive dataset (after dataset redundancy was scaled to 40%).

Bacterial pathogen genus	Number of Virulent proteins
*Escherichia*	222
*Pseudomonas*	144
*Salmonella*	128
*Streptococcus*	73
*Legionella*	85
*Bacillus*	56
*Staphylococcus*	55
*Shigella*	60
*Helicobacter*	53
*Mycobacterium*	49
*Yersinia*	50
*Vibrio*	50

Total	1025

## Results and Discussion

### Algorithm

#### Composition based SVM classifiers

Firstly, we evaluated the SVM classifiers trained and optimized with AAC features – developed with linear, polynomial, sigmoid and Radial Basis Function (RBF) kernels. Each of the kernels was optimized for best performance by changing the kernel parameters (γ, C etc.). We have optimized the SVM classifiers with respect to accuracy and kernel variables. The best C and gamma parameters correspond to maximum accuracy at which the sensitivity and specificity values are nearly equal. For both C and gamma parameters, we have searched a range of 0.005 to 500.

We found that the AAC-SVM classifier optimized with RBF kernel has the highest accuracy (72.1% for γ = 125 and C = 2, Table 2), better than that of SVMs optimized with sigmoid (69.2% accuracy for s = 0.5, C = 75), linear (69.4% accuracy for C = 150) and polynomial (69.6% accuracy for d = 10) kernels. Though, sigmoid, linear and polynomial kernel yielded 69.2%, 69.4% and 69.6% accuracies respectively, the area under ROC plot (ROC described in methods) obtained with sigmoid kernel (0.75), linear kernel (0.75) and polynomial kernel (0.76) were lower as compared to that of RBF kernel (0.79) (Table 3). Table 3 also reveals that the sensitivity and MCC of the AAC-SVM optimized with RBF kernel is much higher as compare to those optimized with other kernels though specificity is marginally lower. Hence, RBF kernel was found to be the most suitable kernel for all the SVM classifiers-trained and tested in the present study. It has been shown that the prediction methods based on compositional features are more accurate than the homology-based searching for example the problems like prediction of functional roles of proteins, secondary structures and subcellular localization [11-14]. In this study too, the different composition-based SVM modules were found to have higher accuracy than that of homology based predictions.

**Table 2:** Performance of SVM classifiers trained with RBF kernel and features based on Amino Acid Composition, PSI-BLAST, PSSM-Profiles and Cascade SVM.

Classifier/training features	Sensitivity (%)	Specificity (%)	Accuracy (%)	MCC
*AAC (A)*	70.0	74.1	72.1	0.44
*Dipeptide Composition(i+1^st^) (B)*	70.0	72.3	71.1	0.42
*Dipeptide Composition (i+2^nd^) (C)*	70.2	73.7	72.0	0.44
*Hybrid1 (A+B+C)*	72.6	75.1	73.9	0.48
*PSI-BLAST search (D)*	52.5	51.7	52.1	----
*Hybrid2 (A+B+C+D)*	79.2	78.8	79.0	0.58
*PSSM (E)*	78.1	78.1	78.1	0.56
*Hybrid3 (A+B+C+D+E)*	79.0	80.1	79.6	0.59
**Cascade **(A+B+C+D+E)	**82.0**	**81.5**	**81.8**	**0.64**

**Table 3:** Area under the ROC curve (AUC) for few optimized SVM classifiers.

Modules developed	Sensitivity (%)	Specificity (%)	Accuracy (%)	MCC	AUC under the ROC curve
AAC (Sigmoid kernel)	63.4	75.0	69.2	0.39	0.75
AAC (Linear kernel)	64.5	74.4	69.4	0.39	0.75
AAC (Polynomial kernel)	63.4	75.8	69.6	0.40	0.76
AAC (RBF kernel)	70.0	74.1	72.1	0.44	0.79
Dipeptide Composition (i+1)	70.0	72.3	71.1	0.42	0.79
Dipeptide Composition (i+2)	70.2	73.7	72.0	0.44	0.79
PSSM-profiles	78.1	78.1	78.1	0.56	0.85
**Cascade classifier**	**82.0**	**81.5**	**81.8**	**0.64**	**0.86**

In order to extract the feature vectors for the terminal segments and central region amino acids, we generated an input vector of 60 dimensions, by calculating the amino acid composition of 20 residues each from the N terminal, C-terminal and the remaining central regions separately. This in turn gives an input vector of 60 dimensions – 20 from N-terminal, 20 from central region and 20 from C-terminal segment. The Figure S1 (see Additional file 1) illustrates the division of sequence into three segments. The idea was based on the assumption that if virulence-determining amino acids are localized in either of the segments, then the chances of generation of a better trained and efficient SVM increase. However, the accuracy of this SVM classifier was not greater than 68.4% for all the SVMs trained using AAC of different lengths of the terminal segments. The performance details of the results obtained for AAC-SVMs based on different lengths of N and C-terminal residues are summarized in the Table S1 (see Additional file 1). Hence we decided to develop SVM classifiers based on features of full-length sequences only.

Further, the AAC analysis of virulent and non-virulent proteins revealed some interesting results. It was observed that for both virulent and non-virulent proteins the average AAC of Leucine, Alanine, Valine and Serine was high, whereas those of Methionine, Histidine, Tryptophan, and Cysteine were amongst the lowest AACs. Furthermore, it was found that residues that have contributed significantly to the development of a successful classification model for virulent and non-virulent proteins are Asparagine, Serine and Alanine (Figure S2, see Additional file 1). In the case of dipeptide composition, the high average composition was observed for dipeptides such as LL, LA, AL, LS, SL, AA, VL, TL, SG, and LV for virulent proteins and for non virulent class the most frequently occurring pairs were – LL, LA, AL, AA, VL, VA, LV, LG, GL, and AG (Figure S4, see Additional file 2). The accuracy of SVM classifier based on dipeptide compositions was 71.1% when trained with the RBF kernel (for γ = 170 and C = 2), which is marginally lower than that of the AAC based SVM module. Though dipeptide composition takes care of the information regarding amino acid composition as well as local order of amino acids, there was no improvement in accuracy over AAC based predictions. It might be due to very low frequency of occurrence of all possible dipeptides in our present dataset. We also developed a SVM modules based on higher order dipeptide compositions (for higher dipeptide compositions, see Figure S4, Additional file 2) to check if the accuracy can be improved further. We found that the higher order dipeptide (*i*+2) composition based SVM classifier was indeed more accurate (72% accuracy, for RBF kernel, γ = 145 and C = 1) than the SVM classifier based on lower order dipeptide composition. The results obtained using traditional and higher order dipeptide composition based SVM modules are summarized in the Table S2 (see Additional file 1). In a nutshell, the composition based SVM classifiers were able to achieve a maximum accuracy of 72.1% (Table 2).

#### Similarity based classifier

The first step in predicting functions of unknown protein sequences is to carry out similarity-based search with the databases of annotated and characterized protein sequences. This approach is successful if a high scoring hit is returned upon database search (usually with a low cut-off E-value). We carried out PSI-BLAST search, using few of the training sequences as query and remaining training sequences as a BLAST database (three iterations with E-value of 0.001). A 5-fold cross-validation was performed such that each of the training sequence was used as a part of a query set as well as a training set sequence. PSI-BLAST correctly predicted 52.5% and 51.7% of virulent and non-virulent proteins respectively, leading to an overall accuracy of 52.1% (Table 2). The detailed results obtained using different iteration values are shown in Table S3 (see Additional file 1). This indicates that similarity-based search alone may not be the best strategy for prediction of different kinds of virulent proteins.

#### Hybrid1 and hybrid2 SVM classifiers

In our attempt to further enhance the prediction accuracy, we developed different hybrid classifiers trained with multiple features, for example: the hybrid1 classifier was trained with a vector of 820 dimensions using individual composition features: 20 for AAC, 400 for dipeptide composition, and 400 for higher order dipeptide composition. This classifier was able to predict virulent proteins with an accuracy of 73.9% (for RBF kernel, γ = 50 and C = 1), around 2% more than that of the SVM classifiers based on AAC and higher order dipeptide composition features.

Hybrid2 SVM classifier was trained with composition-based (hybrid1) and similarity-search based features. SVM training with similarity-search based results, together with compositional features enhanced the accuracy from 73.9% to 79% (for γ = 50 and C = 1), approximately 5% improvement over that of the hybrid1 classifier (Table 2). Therefore, similarity-based search combined with composition-based features yielded higher accuracy.

#### PSSM based SVM classifier

The features based on multiple sequence alignments of protein sequences have been successfully applied to improve accuracy of prediction algorithms for secondary structure, solvent accessibility and subcellular localization [14-17]. The basis of the success of the alignment based methods lies in the fact that during protein evolution, the amino acid residues with similar physico-chemical properties tend to be conserved due to selective pressure. PSI-BLAST PSSM profiles as a SVM training feature have been successfully applied for the prediction of solvent accessibility [16]. We found that use of 400 dimensional input vector of PSSM matrix without normalization, failed to classify virulent and non-virulent proteins with significant accuracy. This may be due to presence of highly divergent scoring values in the generated matrices. Hence, we scaled down each matrix element to the range between 0–1 by using a sigmoid function for the normalization of matrices. The highest accuracy of SVMs trained on normalized PSSM profiles was 78.1% (RBF kernel, for γ = 22 and C = 5), significantly better than any of the SVM classifiers based on individual features, developed in the present study (Table 2). Significantly, the classifier was 4% more accurate than the hybrid1 classifier. Moreover, the classifier performance is better as compared to the similarity based search (PSI-BLAST) and at par with that of the hybrid2 classifier.

#### Hybrid3 SVM classifier

Hybrid3 classifier was trained with hybrid2 features and features based on PSSM profiles. The accuracy of optimized hybrid3 classifier was 79.6% (for γ = 25 and C = 2), i.e. around 1% improvement over that of the PSSM based SVM classifier (Table 2) and comparable to that of the hybrid2 classifier. Limitation in further improvement in accuracy may be attributable to noise produced by a relatively larger number of input features (i.e. a vector of greater than 1200 dimensions) and diverse nature of virulent proteins.

#### Cascade SVM classifier

**Figure 1:**
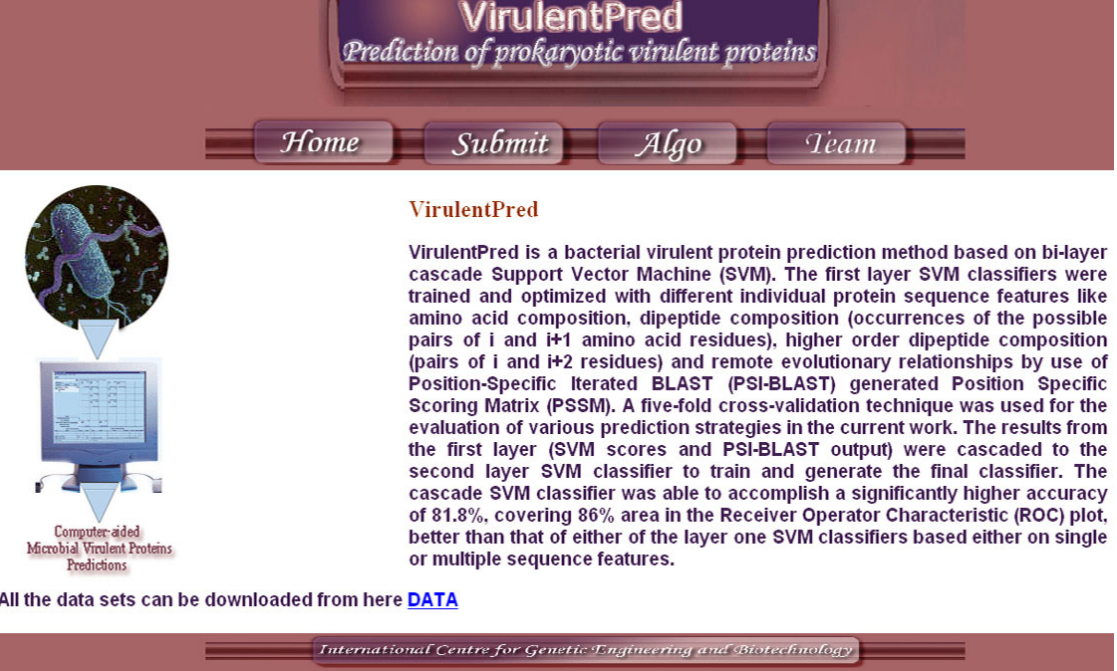
**VirulentPred web server**. The bi-layer Cascade SVM is used as default method for VirulentPred predictions (at the default threshold value of 0.0) as it was found to be most accurate after evaluation of different SVM classifiers developed in the study.

Evaluations of cascade classifiers based on different hybrid features revealed that the best classifier is the one trained with hybrid3 features. The accuracy of the optimized cascade SVM classifier was 81.8% (for γ = 5 and C = 0.5), which is significantly higher than that of all the individual and hybrid SVM classifiers developed in the study (Table 2). The detailed results obtained using the cascade SVM module at different threshold values is shown in Table S4. The classifier ROC curve analysis revealed that the area under curve (AUC): 0.86 for the classifier was greater than that of other non-cascade classifiers (Table 3). Additionally, we also compared the performances of cascade modules of all the hybrids with accuracy greater than 79.6 (Table S5, Additional file 1), and found the performance of the cascade module of hybrid3 features to be the best. Hence the cascade SVM classifier was chosen for further validation with the independent datasets sequences and generating the VirulentPred web server (Figure 1).

### Testing

#### Prediction performance for independent datasets

An accuracy of 81.9% was achieved on an independent dataset I using the cascade SVM classifier (henceforth referred to as VirulentPred) (Table 4). VirulentPred correctly predicted 33 virulent (82.5%) and 35 non-virulent (81.4%) proteins. Independent dataset II was used to check if VirulentPred can be used to predict virulent proteins in the proteomes of organisms, the sequences of which were not used for SVM training. Out of 284 independent dataset II protein sequences, 228 proteins were correctly predicted by VirulentPred, leading to an overall accuracy of 80.3%. Further, the prediction accuracies for the *Bordetella*, *Campylobacter, Haemophilus*, *Listeria and Neisseria *protein sequences were calculated to be 79.6%, 79.8%, 82.9%, 81.3% and 77.6%, respectively. The prediction accuracies discussed above indicate that VirulentPred may be used as a general virulent protein sequence prediction method.

**Table 4:** Performance of cascade SVM classifier for the independent dataset I and II sequences.

	***Independent Dataset I***	***Independent Dataset II***					
		
		*Bordetella*	*Campylobacter*	*Listeria*	*Neisseria*	*Haemophilus*	*Overall*
		
**Sensitivity (%)**	82.5 (33)	77.8 (21)	79.5 (31)	73.3 (11)	84.0 (21)	85.7 (30)	80.9 (114)
**Specificity (%)**	81.4 (35)	81.5 (22)	80.0 (32)	88.2 (15)	70.8 (17)	80.0 (28)	79.7 (114)
**Accuracy (%)**	**81.9 (68)**	79.6 (43)	79.8 (63)	81.3 (26)	77.6 (38)	82.9 (58)	**80.3 (228)**

Values in parenthesis correspond to the number of true hits obtained.

#### Prediction performance for eukaryotic proteins

Finally, we also evaluated the performance of VirulentPred for eukaryotic protein sequences. The eukaryotic virulent proteins were mainly neurotoxins and the test sequences (both virulent and non-virulent) were randomly selected from the database used to develop NTXpred method [18]. Since, the method was purely trained on prokaryotic protein sequences; a lower accuracy was expected for eukaryotic proteins. Unexpectedly, the method was able to correctly predict 48 query virulent proteins as virulent, with a sensitivity of 96%. However, VirulentPred was unable to predict non-virulent proteins. Out of the 50 randomly selected non-virulent proteins from the NTXpred dataset, only 8 proteins (16%) were correctly predicted, giving a high percent of false-positives. Hence, an overall performance (56%) of the classifier for the prediction of eukaryotic virulent protein sequences is very poor. The poor performance of VirulentPred for eukaryotic sequences may be due to amino acid compositional differences with the virulent proteins of prokaryotic origin. As expected, we also observed differences in AACs of eukaryotic and prokaryotic virulent proteins. It was found that in the eukaryotic virulent proteins, most frequently occurring amino acids residues are Cysteine, Glycine, Lysine, Leucine, and Serine, whereas Lysine, Alanine, and Serine in bacterial virulent proteins. The dipeptides such as GY, YC, CK, SG, GK, KK, and KC were more abundant in eukaryotic virulent proteins, and that in the case of bacterial proteins – LL, LA, AL, LS, SL, AA, VL, and TL. The eukaryotic virulent and non virulent datasets may be downloaded from VirulentPred web server site.

#### Predictions for bacterial proteomes

**Figure 2:**
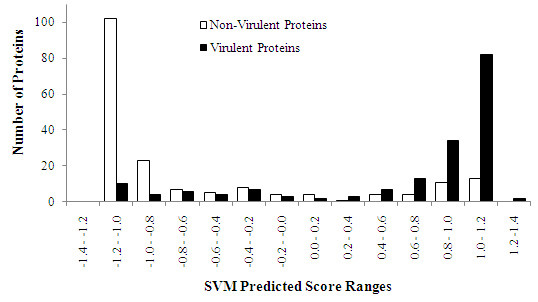
**VirulentPred predictions in the various ranges of SVM scores**. For generating the plot, 367 sequences of both the independent datasets (consisting of 181 virulent and 186 non-virulent proteins) were classified using VirulentPred. The number of false positive prediction was very high at a threshold SVM scores in the range 0.8 to 1.1. Hence, to reduce false positive prediction, a stringent criterion of threshold value of ≥1 was used for the annotation of complete proteomes of pathogens.

We also tested VirulentPred predictions on complete proteomes of several bacterial pathogens. To perform the tests, we decided to use a higher threshold (higher than the default threshold used for training and testing SVMs) to minimize false positive hits, as these were blind tests, without any supportive evidence for the query proteins to be virulent- for example- to be surface bound, secreted or released. To arrive at a reasonable stricter threshold value, we distributed the independent datasets proteins (Independent dataset I and II, combined) according to their SVM scores into different ranges between -1.4 to 1.4 (Figure 2). The distribution shows that several non-virulent proteins (false positives) fall up to the range corresponding to the SVM score of 1. However, it may be seen that for a threshold of ≥1.0, most of the true positive hits are captured and the false positive predictions are minimum, hence we decided to use a threshold of ≥1.0 for whole genome predictions. A similar analysis for the training dataset (2055 sequences) also yielded similar results, supporting the choice for a higher threshold (Figure S3, see Additional file 1), a summary of scores in different ranges for the independent dataset sequences and training dataset sequences is available in the Table S6 (see Additional file 1).

**Figure 3:**
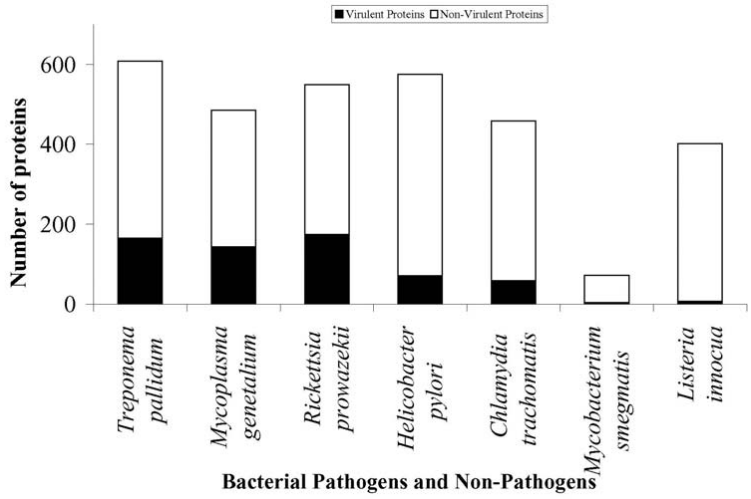
**VirulentPred predictions for different proteomes**. The plot depicts the number of proteins predicted to be virulent (at a higher threshold value, ≥1) in proteomes of 7 different bacteria.

Using the higher threshold of SVM score, we started our search with the protein sequences of the smallest forms of the Monera kingdom-*Mycoplasma genetalium*, a parasitic bacterium colonizing in genital and respiratory tracts of primates. Mycoplasma genetalium is of special interest to the developmental biologists as it is the organism with the smallest genome, next only to that of viruses. Out of 485 protein sequences of *Mycoplasma genetalium*, VirulentPred was able to classify 295 sequences, (60.8% of the total proteome) as virulent on the basis of SVM predicted scores at the threshold value of 0.0. However, at a threshold ≥1.0, 29.5% of sequences were predicted as virulent. In addition, we also checked the performance of our method for proteomes of *Chlamydia trachomatis *(458 sequences), *Rickettsia prowazekii *(549 sequences), *Helicobacter pylori *(575 sequences), and *Treponema pallidum *(608 sequences). The prediction summary obtained for proteomes of the 5 pathogens are shown in Figure 3. Besides, we also tested VirulentPred method on the complete proteomes of two non-pathogenic bacteria such as *Mycobacterium smegmatis *(72) and *Listeria innocua *(402) to establish the reliability of VirulentPred method. The outputs show that the chances of false prediction are very less for the prediction of virulent proteins at higher threshold, hence increasing the reliability.

Several protein sequences were predicted to be virulent with high scores. High scoring proteins include a host of experimentally verified virulent proteins and hypothetical proteins in several bacterial proteomes. The examples of the latter include the following SWISS-PROT entries of *Rickettsia prowazekii *proteins: Y169_RICPR (1.2), Y192_RICPR (1.3), Y222_RICPR (1.2), Y244_RICPR (1.2), Y867_RICPR (1.2).

### Implementation

The SVM classifiers developed in the study have been implemented as World Wide Web Server – VirulentPred using CGI/PERL scripts (Figure 1). Though the server provides options to select different classifiers and threshold values, a default prediction is performed with the bi-layer cascade SVM classifier at a threshold value of 0.0. The server accepts input protein sequences in FASTA format.

### Comparison with other servers

To the best of our knowledge, there is no report of a computational method to classify protein sequences into virulent and non-virulent. However, recently two methods: SPAAN [9] and VICMpred [19] have been reported which predict specific virulent proteins. VICMpred classifies bacterial proteins into 4 different functional classes: virulence factors, information molecule, cellular process and metabolism molecule (with an accuracy of 70.75%). However, the method is based on training with 70 gram-negative bacterial virulence protein sequences whereas VirulentPred is trained with 1025 diverse virulent protein sequences from different bacteria. Hence, it is not justified to compare VirulentPred with the VICMpred method. On the other hand, SPAAN is a neural network based method for prediction of adhesins only. However, we have run VirulentPred on SPAAN dataset of 469 adhesins and 703 non-adhesins proteins. We found that out of 469 adhesins, VirulentPred was able to predict 412 proteins as virulent (87.8% correct predictions). This may be due to the fact that VirulentPred has been developed as a general virulent protein prediction method, where SVMs were trained with diverse features of different kinds of virulent proteins. As a result, there could be decay in the signals related to a particular class of virulent proteins. However, out of the 703 non-adhesin protein sequences, only 410 sequences were correctly predicted to be non-virulent. This was mainly due to the fact that the SPAAN training dataset includes several archaebacterial, viral and yeast non virulent proteins. However, VirulentPred was developed only with bacterial sequences; hence the method was not very efficient in prediction of viral and eukaryotic protein sequences. Intriguingly, out of 364 SPAAN non virulent protein sequences of bacterial and archaebacterial origin, VirulentPred correctly predicted 263 protein sequences (72.3%).

## Conclusion

VirulentPred classifies bacterial virulent sequences from non virulent proteins with an accuracy of 81.8%. We have demonstrated that VirulentPred efficiently classifies sequences not used in the training, including the ones from the organisms independent of the study. For the whole proteome runs, the VirulentPred prediction efficiency is better when predictions are run with higher threshold. The VirulentPred predicts virulent proteins irrespective of the subclass or specific molecular function hence the method may be used as general prediction method for virulent protein sequences from prokaryotic genomes.

## Methods

### Generation of training dataset

The bacterial virulent protein sequences were retrieved from the SWISS-PROT [20] and VFDB (an integrated and comprehensive database of virulence factors of bacterial pathogens, [21]). SWISS-PROT sequences were retrieved using keywords such as virulence, adhesin, adhesion, adherence, toxin, invasion, capsule and other terms related to virulence factors. The VFDB and SWISS-PROT sequences were screened strictly in order to obtain a high quality dataset. First, the sequences were filtered to remove entries annotated as "Probable", Putative", "By similarity", "Fragments" "Hypothetical", "Unknown" and "Possible". The filtering yielded 1756 annotated virulent protein sequences (henceforth referred to as positive dataset).

For training with non-virulent protein sequences, we selected 3000 annotated protein sequences of bacterial enzymes and other non-virulent proteins from SWISS-PROT database (these sequences are henceforth referred to as negative dataset). The negative dataset sequences were mainly chosen from the bacterial proteomes, the virulent protein sequences of which are included in the positive dataset.

### Reducing redundancy of datasets

Next step in the refinement of dataset is to reduce similarity present between sequences. We used PROSET [22] to scale the redundancy in positive and negative dataset sequences such that no two sequences were more than 40 percent similar. PROSET yielded a non-redundant dataset of sequences, out of which 1206 sequences were found to be virulent (positive dataset) sequences. Out of these sequences, we selected out 141 sequences belonging to 5 different organisms to make the independent dataset II. From the remaining 1065 sequences, 40 were randomly picked up to make the independent dataset I. Hence we used 1025 positive sequences as shown in the Table 1 and 1030 negative sequences from bacterial proteomes to make our final negative dataset. Hence, the final non-redundant dataset (2055 sequences) consists of 1025 virulent and 1030 non-virulent sequences. This dataset was used for training different SVM classifiers developed and discussed in the study is freely available at VirulentPred web server site.

### Generation of datasets for blind tests

#### Independent dataset I

Independent dataset I consist of 83 SWISS-PROT sequences (40 virulent and 43 non-virulent protein sequences), randomly selected from non-redundant positive and negative datasets (henceforth referred to as independent dataset I). The redundancy of the independent dataset I sequences was scaled, such that no two dataset sequences were more than 40% similar. The independent dataset I sequences were used to evaluate the unbiased performance of different classifiers developed in the present study.

#### Independent dataset II

Sequences of a few organisms were excluded from the positive non-redundant training dataset to constitute a positive independent dataset II. This was done to gauge the classifier prediction efficiency for the sequences of the organisms, which were not represented in the training dataset. Similarly, random non-virulent sequences from these organisms were included in the negative independent dataset II. The prediction accuracy for this dataset reflects the general applicability of the classifiers for the sequences from different Monera kingdom organisms. The dataset consists of 141 virulent and 143 non-virulent sequences from the bacterial pathogens-*Campylobacter *(39 virulent and 40 non-virulent protein sequences), *Neisseria *(25 virulent and 24 non-virulent), *Bordetella *(27 virulent and 27 non-virulent sequences), *Haemophilus *(35 virulent and 35 non-virulent) and *Listeria *(15 virulent and 17 non-virulent).

#### Test dataset of different proteomes

The efficiency of the methods was also tested for the complete protein sequences of different bacterial pathogens and non-pathogens such as *Mycoplasma genetalium *(485 sequences), *Chlamydia trachomatis *(458 sequences), *Rickettsia prowazekii *(549 sequences), *Helicobacter pylori *(575 sequences), *Treponema pallidum *(608 sequences), *Mycobacterium smegmatis *(72 sequences) and *Listeria innocua *(402 sequences). All the protein sequences of the organisms discussed above were retrieved from the SWISS-PROT database.

### SVM training

The SVM classifiers were developed using SVM^light ^package [23]. The software implements machine learning with a number of optimization parameters and kernels (e.g. linear, sigmoid, polynomial, and radial basis function (RBF) for training. The choices of kernel and parameters were optimized for best performance using cross validation techniques. Implementation of SVM learning requires input of fixed length patterns; therefore it becomes mandatory to convert the features corresponding to each of the 20 amino acids in protein sequences (of variable length) into a fixed length feature input. A brief description of the input features and methods to convert them into fixed length features are described below.

### Composition based SVM classifiers

AAC is the fraction of each of the 20 amino acids present in a protein sequence. The calculation of fraction of amino acids in a protein sequence generates a vector of 20 dimensions.

Dipeptide composition is the number of occurrence of a dipeptide divided by the number of possible dipeptides. The advantage of dipeptide composition over AAC is that the former encapsulates information about the fractions of amino acids as well as its local order in a protein sequence. Calculation of dipeptide frequencies yields a training vector of 400 dimensions.

### PSI-BLAST similarity based search

We evaluated PSI-BLAST search [8] to find virulent protein sequences using the training database sequences. The choice of PSI-BLAST was made instead of the standard BLAST, as PSI-BLAST performs remote similarity search. Three iterations of PSI-BLAST search were performed with a cut-off E value of 0.001. The results of the PSI-BLAST search were also evaluated using five-fold cross-validation technique. Here, 4 sets were made BLAST database and the remaining fifth set sequences were used to query the database, this cycle was repeated 5 times with different combinations of datasets so that each of the sequences in the datasets is used to query the BLAST database of the sequences belonging to remaining datasets.

### Training features based on PSSM

We also used PSI-BLAST generated PSSM profiles as a training feature. In this case, PSI-BLAST iterative search was performed against the non-redundant NCBI database, with a cut-off E-value of 0.001. In each of the 3 iterations, a profile or PSSM (Position Specific Scoring Matrix) is generated from a multiple alignment of the high scoring hits by calculating position specific scores for each position in alignments. The PSSM generated in each step is used to perform next iterative search, thereby increasing the sensitivity of the search in each step. After three iterations, PSI-BLAST generates a PSSM having the highest score. The matrix contains 20 times N elements, where N is the length of the target sequence, and each element represents the frequency of occurrence of each of the 20 amino acids at a particular position in the alignment. Subsequently, the final PSSM was normalized using a sigmoid function (Equation 1) by which each matrix element *f(x) *was scaled to a range 0–1.

(1) $$f(x)=\;\frac{1}{1+e^{-x}}$$

**Figure 4:**
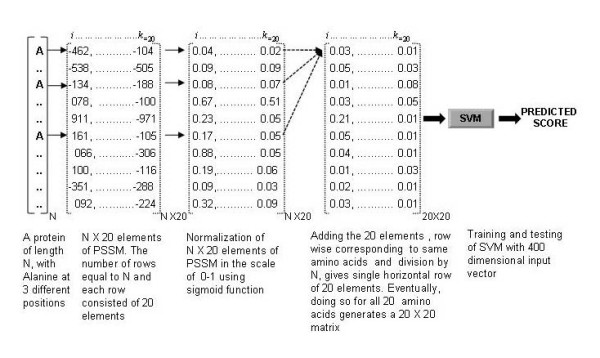
**Conversion of PSSM into training vectors**. The steps used to convert PSSM profiles generated by PSI-BLAST into a training vector of 400 dimensions.

To make a SVM input of fixed length, we summed up all the rows in the PSSM corresponding to the same amino acid in the sequence, followed by division of each element by the length of the sequence. The steps used to generate an input of 400 dimensions are shown in Figure 4.

### Hybrid SVM Classifiers

A hybrid SVM classifier was trained using combination of features-AAC, dipeptide and higher order dipeptide (henceforth referred to as hybrid1). In developing hybrid1, the SVM classifiers were trained with an input vector of 820 dimensions: 20 for AAC, 400 each for dipeptide and higher order dipeptide compositions.

In hybrid2 SVM classifier, similarity-search based features were incorporated along with composition based-SVM classifiers. Hybrid2 SVM classifiers were trained with an input vector of 823 dimensions: 820 from hybrid1 classifier and 3 from PSI-BLAST output. PSI-BLAST output was converted to binary variables using the notation: (1 0 0) for virulent protein, (0 1 0) for non-virulent protein, and (0 0 1) for unknown or proteins without any match in the database.

The hybrid3 SVM classifier encapsulated a wider range of features from a protein sequence. The hybrid3 classifier was trained using 820 dimensions of all possible compositions based features, 3 from similarity-based results and 400 of PSSM profiles, i.e. a vector of 1223 dimensions.

### Cascade SVM classifier

**Figure 5:**
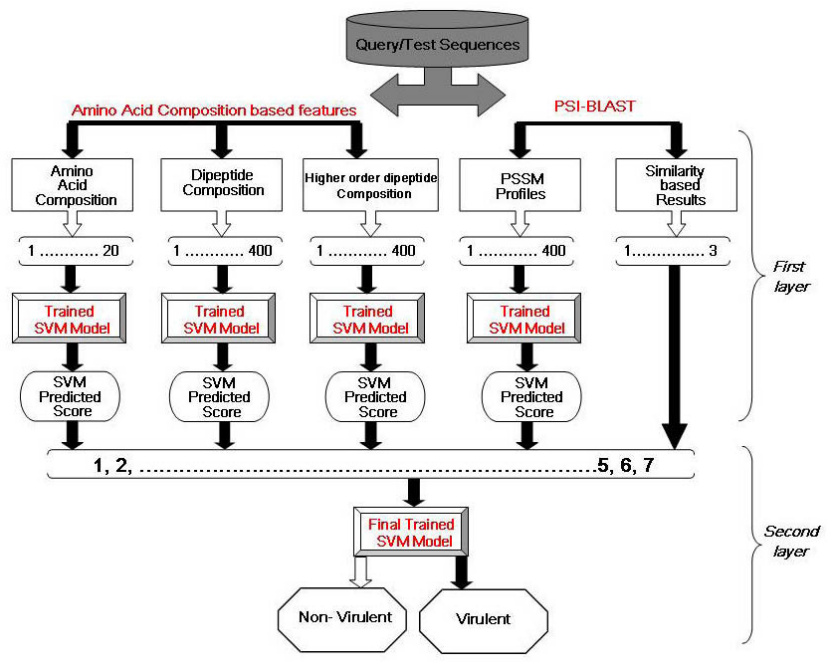
**Schema of the bi-layer cascade SVM module**. The SVM classifier was the most efficient classifier developed in the study.

Classification efficiency of machine learning techniques is diminished by noise in large and complex datasets. However, this problem may be overcome by the layered SVM [24] in certain cases. To explore the effectiveness of this strategy for the training dataset used in the study, we generated a bi-layered cascade SVM classifier. The first layer of the cascade SVM consists of classifiers based on individual protein features discussed earlier (Figure 5). The second layer was trained with the binary scores of the output generated by 5 best classifiers in the first layer. The second layer SVM was trained with a vector of 7 dimensions (1 for AAC, 1 for dipeptide composition, 1 for higher order dipeptide composition, 1 for PSSM and 3 for PSI-BLAST results). Hence, the second layer SVM learns from the first layer classifiers and PSI-BLAST results to generate a final cascade SVM classifier.

### SVM evaluation Parameters

Five-fold cross-validation technique was used to judge the performance of SVM classifiers developed in the present study. The training dataset of virulent and non-virulent proteins was divided randomly into five subsets containing equal number of both types of proteins. The classifiers were trained on four sets and performance was assessed on the fifth set. This process was iterated five times so that each set was used as a training and test dataset. The final performance of a classifier is the averaged performance using all the five test sets.

Performance of a prediction method may be assessed either by threshold independent or threshold dependent parameters, each of the parameters have few limitations. We calculated four threshold dependent parameters; sensitivity, specificity, accuracy and Matthews Correlation Coefficient (MCC) to perform cross-validation as well as checking the prediction accuracy for the independent dataset sequences. A brief descriptions of the parameters used in the study are discussed below.

Sensitivity is the percentage of virulent proteins correctly predicted as virulent (*p*) as shown in the Equation 2 (*u *is the number of under predicted sequences).

(2) $$\text{Sensitivity}=\frac{p}{p+u}$$

Specificity is the percentage of non-virulent proteins correctly predicted as non-virulent (*n*), Equation 3 (*o *is the number of over-predicted sequences)

(3) $$\text{Specificity}=\frac{n}{n+o}$$

Accuracy is the percentage of correctly predicted virulent and non-virulent proteins from total number of protein sequences (*t*). (Equation 4)

(4) $$\text{Accuracy}=\frac{p+n}{t}$$

MCC equal to 1 is regarded as a perfect prediction, whereas 0 is for a completely random prediction. (Equation 5)

(5) MCC  = pn−ou(p+o) (p+u) (n+o) (n+u)

Threshold dependent classifications are useful for decision making, however, these fail to reflect the performance of classifiers independent of thresholds. There are several measures to calculate threshold independent performance, one such measure is a ROC plot. ROC plots are obtained by plotting all sensitivity values (true positive fraction) on the y-axis against their equivalent (1-specificity) values (false positive fraction) on the x-axis. The area under the ROC curve (AUC) is considered to be an important index because it provides a single measure of overall threshold independent accuracy. If the value is 0.5 or less, the scores for two classes do not differ much, while a score of 1.0 indicates no overlap in the distributions of the group scores.

## Availability and requirements

Project name: Prediction of bacterial virulent proteins;

Project home page: http://bioinfo.icgeb.res.in/virulent/;

Operating system(s): Platform independent;

Programming language: PERL, CGI-PERL;

License: None;

Any restrictions to use by non-academics: No restrictions

## List of abbreviations

SVM, Support Vector Machine; PSSM, Position specific scoring matrix; AAC, Amino acid compositions; RBF, Radial basis function; AUC, Area under curve; ROC, Receiver Operator Characteristic; MCC, Matthews Correlation Coefficient.

## Authors' contributions

AG carried out the data mining, trained SVM, data analysis, interpretation and wrote computer programs. DG and AG wrote the manuscript and developed the web server. DG coordinated the project. Both authors read and approved the final manuscript.

## Supplementary Material

Additional file 1 Six tables (Table S1–S6) giving: effect of compositions derived from different N and C-terminal lengths on the performance of SVM based module (Table S1), the results obtained using traditional and higher order dipeptide composition based SVM modules (Table S2), the performance of PSI-BLAST searches using different iteration values (Table S3), detailed results obtained for Cascade SVM module at different threshold values (Table S4), the parameters of optimized SVMs obtained using different individual features and its combination (Table S5), and the distribution of 2055 proteins and Independent datasets (combined) into different ranges in the scale of -1.4 to 1.4 according to their SVM predicted scores for cascade SVM module (Table S6). The file also contains three figures (Figure S1–S3), giving: Schema illustrating the strategy to calculate AAC of N, C and middle regions of a protein (Figure S1), The difference in average amino acid composition for virulent and non-virulent proteins (Figure S2), The number of proteins predicted in different ranges of SVM scores using complete 2055 sequences (Figure S3). Click here for file

Additional file 2 One worksheet and chart (Figure S4), representing dipeptide frequency data of i+1, i+2 and i+3 dipeptides in virulent and non-virulent proteins and its distribution. Click here for file
