# Supplementary material for: VirulentPred: a SVM based prediction method for virulent proteins in bacterial pathogens
Source: BMC Bioinformatics. 2008 Jan 28;9:62. doi: 10.1186/1471-2105-9-62 (PMC2254373; doi:10.1186/1471-2105-9-62)

**ADDITIONAL MATERIAL file 1**

**Table S1. Effect of compositions derived from different N and C-terminal lengths on the performance of SVM based module**

| ***Terminal lengths*** | ***Sensitivity (%)*** | ***Specificity (%)*** | ***Accuracy (%)*** | ***MCC*** | ***SVM parameters for kernel=RBF*** |
| --- | --- | --- | --- | --- | --- |
| 10 | 65.0 | 68.8 | 66.9 | 0.34 | γ=1, C=20 |
| 15 | 66.1 | 67.4 | 66.8 | 0.34 | γ=15, C=0.5 |
| 20 | 65.6 | 68.9 | 67.3 | 0.35 | γ=2, C=20 |
| **25** | **69.2** | **67.7** | **68.4** | **0.37** | **γ=20, C=2** |
| 30 | 64.1 | 69.9 | 67.0 | 0.34 | γ=3, C=25 |

**Table S2. The results obtained using traditional and higher order dipeptide composition based SVM modules**

| **Dipeptide Types** | **Sensitivity *(%)*** | **Specificity *(%)*** | **Accuracy *(%)*** | **MCC** | **SVM parameters for kernel=RBF** |
| --- | --- | --- | --- | --- | --- |
| *i*+1 | 70.0 | 72.3 | 71.1 | 0.42 | γ=170, C=2 |
| *i*+2 | 70.2 | 73.7 | 72.0 | 0.44 | γ=145, C=1 |
| *i*+3 | 69.4 | 71.9 | 70.7 | 0.41 | γ=175, C=1 |
| *i*+4 | 69.9 | 71.1 | 70.5 | 0.41 | γ=200, C=2 |

**Table S3. The performance of PSI- BLAST searches using different iteration values.**

| **Number of iterations** | **Sensitivity (%)** | **Specificity (%)** | **Accuracy (%)** | **No. of proteins with no hits** |
| --- | --- | --- | --- | --- |
| 1 | 50.7 | 50.8 | 50.8 | 809 |
| 2 | 50.0 | 50.5 | 50.2 | 806 |
| 3 | 52.5 | 51.7 | 52.1 | 766 |
| 4 | 52.8 | 51.5 | 52.1 | 766 |

**Table S4. Detailed results obtained for Cascade SVM module at different threshold values.**

| **Threshold values** | **Sensitivity** | **Specificity** | **Accuracy** | **MCC** | **TP** | **TN** | **FP** | **FN** |
| --- | --- | --- | --- | --- | --- | --- | --- | --- |
| -1.3 | 100.0000 | 0.0971 | 49.9270 | 0.02 | 1025 | 1 | 1029 | 0 |
| -1.2 | 100.0000 | 0.2913 | 50.0243 | 0.04 | 1025 | 3 | 1027 | 0 |
| -1.1 | 98.4390 | 6.7961 | 52.5061 | 0.13 | 1009 | 70 | 960 | 16 |
| -1.0 | 92.8780 | 56.2136 | 74.5012 | 0.53 | 952 | ***579*** | 451 | ***73*** |
| -0.9 | 89.5610 | 68.5437 | 79.0268 | 0.59 | 918 | 706 | 324 | 107 |
| -0.8 | 88.4878 | 71.1650 | 79.8054 | 0.61 | 907 | 733 | 297 | 118 |
| -0.7 | 87.4146 | 73.1068 | 80.2433 | 0.61 | 896 | 753 | 277 | 129 |
| -0.6 | 86.4390 | 74.4660 | 80.4380 | 0.61 | 886 | 767 | 263 | 139 |
| -0.5 | 85.4634 | 76.2136 | 80.8273 | 0.62 | 876 | 785 | 245 | 149 |
| -0.4 | 84.3902 | 77.7670 | 81.0706 | 0.62 | 865 | 801 | 229 | 160 |
| -0.3 | 83.9024 | 78.9320 | 81.4112 | 0.63 | 860 | 813 | 217 | 165 |
| -0.2 | 83.5122 | 79.8058 | 81.6545 | 0.63 | 856 | 822 | 208 | 169 |
| -0.1 | 82.8293 | 80.4854 | 81.6545 | 0.63 | 849 | 829 | 201 | 176 |
| ***0.0*** | ***82.0488*** | ***81.4563*** | ***81.7518*** | ***0.64*** | ***841*** | ***839*** | ***191*** | ***184*** |
| 0.1 | 80.4878 | 82.0388 | 81.2652 | 0.63 | 825 | 845 | 185 | 200 |
| 0.2 | 79.9024 | 82.6214 | 81.2652 | 0.63 | 819 | 851 | 179 | 206 |
| 0.3 | 78.9268 | 82.9126 | 80.9246 | 0.62 | 809 | 854 | 176 | 216 |
| 0.4 | 78.3415 | 83.9806 | 81.1679 | 0.62 | 803 | 865 | 165 | 222 |
| 0.5 | 77.1707 | 84.7573 | 80.9732 | 0.62 | 791 | 873 | 157 | 234 |
| 0.6 | 76.0000 | 85.4369 | 80.7299 | 0.62 | 779 | 880 | 150 | 246 |
| 0.7 | 74.7317 | 86.0194 | 80.3893 | 0.61 | 766 | 886 | 144 | 259 |
| 0.8 | 72.0000 | 87.2816 | 79.6594 | 0.60 | 738 | 899 | 131 | 287 |
| 0.9 | 68.4878 | 88.7379 | 78.6375 | 0.58 | 702 | 914 | 116 | 323 |
| 1.0 | 55.4146 | 91.5534 | 73.5280 | 0.50 | 568 | 943 | 87 | 457 |
| 1.1 | 11.6098 | 96.9903 | 54.4039 | 0.17 | 119 | 999 | 31 | 906 |
| 1.2 | 2.9268 | 99.4175 | 51.2895 | 0.09 | 30 | 1024 | 6 | 995 |
| 1.3 | 0.3902 | 100.0000 | 50.3163 | 0.04 | 4 | 1030 | 0 | 1021 |

**Table S5. The parameters of optimized SVMs obtained using different individual features and its combination**

| **Model Types** | **Sensitivity** | **Specificity** | **Accuracy** | **MCC** | **Parameters for RBF kernel** |
| --- | --- | --- | --- | --- | --- |
| *Individual modules* | | | | | |
| AAC (A) | 70.0 | 74.1 | 72.1 | 0.44 | γ=125, C=2 |
| i+1 dipeptide (B) | 70.0 | 72.3 | 71.1 | 0.42 | γ=170, C=2 |
| i+2 dipeptide (C) | 70.2 | 73.7 | 72.0 | 0.44 | γ=145, C=1 |
| PSI-BLAST (D) | 52.5 | 51.7 | 52.1 | ----- |  |
| PSSM (E) | 78.1 | 78.1 | 78.1 | 0.56 | γ=22, C=5 |
| *Dihybrids* | | | | | |
| A+B | 74.0 | 74.5 | 74.2 | 0.48 | γ=75, C=1 |
| A+C | 72.7 | 74.1 | 73.4 | 0.47 | γ=80, C=1 |
| A+D | 79.8 | 77.4 | 78.6 | 0.57 | γ=160, C=0.05 |
| A+E | 78.1 | 78.1 | 78.2 | 0.56 | γ=20, C=4 |
| B+C | 72.8 | 71.8 | 72.3 | 0.45 | γ=100, C=2 |
| B+E | 79.7 | 77.9 | 78.8 | 0.58 | γ=25, C=3 |
| B+D | 77.8 | 79.1 | 78.4 | 0.57 | γ=125, C=0.05 |
| C+E | 78.8 | 78.3 | 78.6 | 0.57 | γ=25, C=3 |
| C+D | 77.9 | 80.4 | 79.1 | 0.58 | γ=100, C=2 |
| E+D | 80.8 | 80.9 | 80.8 | 0.62 | γ=25, C=3 |
| Cascade of E+D | 78.6 | 82.5 | 80.6 | 0.61 | γ=25, C=4 |
| *Trihybrids* | | | | | |
| A+B+C | 72.6 | 75.1 | 73.9 | 0.48 | γ=50, C=1 |
| A+B+E | 79.5 | 77.7 | 78.4 | 0.57 | γ=25, C=3 |
| A+B+D | 80.2 | 77.6 | 78.9 | 0.58 | γ=75, C=7 |
| A+C+E | 78.3 | 79.4 | 78.9 | 0.58 | γ=20, C=2 |
| A+C+D | 80.4 | 79.1 | 79.8 | 0.60 | γ=60, C=2 |
| Cascade of A+C+D | 81.3 | 79.4 | 80.3 | 0.61 | γ=38, C=2 |
| A+D+E | 81.3 | 81.2 | 81.2 | 0.62 | γ=13, C=3 |
| Cascade of A+D+E | 81.0 | 82.3 | 81.7 | 0.63 | γ=15, C=17 |
| B+C+E | 79.5 | 77.6 | 78.5 | 0.57 | γ=20, C=10 |
| B+C+D | 77.1 | 80.7 | 78.9 | 0.58 | γ=50, C=1 |
| B+D+E | 81.4 | 81.5 | 81.4 | 0.63 | γ=20, C=2 |
| Cascade of B+D+E | 80.9 | 81.5 | 81.2 | 0.62 | γ=12, C=1 |
| C+D+E | 81.5 | 81.2 | 81.3 | 0.63 | γ=20, C=2 |
| Cascade of C+D+E | 80.7 | 81.8 | 81.3 | 0.63 | γ=17, C=5 |
| *Tetrahybrids* | | | | | |
| A+B+C+D | 79.2 | 78.8 | 79.0 | 0.58 | γ=50, C=2 |
| A+B+C+E | 79.4 | 78.2 | 78.8 | 0.58 | γ=20, C=3 |
| B+C+D+E | 81.2 | 81.5 | 81.3 | 0.63 | γ=15, C=3 |
| Cascade of B+C+D+E | 81.7 | 81.2 | 81.4 | 0.63 | γ=12, C=4 |
| A+C+D+E | 81.3 | 81.2 | 81.2 | 0.62 | γ=13, C=3 |
| Cascade of A+C+D+E | 81.9 | 81.5 | 81.7 | 0.63 | γ=39, C=0.5 |
| A+B+D+E | 81.9 | 80.3 | 81.1 | 0.62 | γ=20, C=2 |
| Cascade of A+B+D+E | 82.0 | 81.6 | 81.8 | 0.64 | γ=10, C=2 |
| *Pentahybrid* | | | | | |
| A+B+C+D+E | 79.0 | 80.1 | 79.6 | 0.59 | γ=20, C=2 |
| Cascade of A+B+C+D+E | 82.0 | 81.5 | 81.8 | 0.64 | γ=15, C=0.5 |

**Table S6. The distribution of 2055 proteins and Independent datasets (combined) into different ranges in the scale of –1.4 to 1.4 according to their SVM predicted scores for cascade SVM module.**

| **Ranges of Predicted SVM scores** | **For Complete dataset of 2055 sequences** | | **For combined independent dataset of 367 sequences** | |
| --- | --- | --- | --- | --- |
|  | **Number of Non-Virulent Proteins** | **Number of Virulent Proteins** | **Number of Non-Virulent Proteins** | **Number of Virulent Proteins** |
| -1.4 - -1.2 | 3 | 0 | 0 | 0 |
| -1.2 - -1.0 | 576 | 73 | 102 | 10 |
| -1.0- -0.8 | 154 | 45 | 23 | 4 |
| 0.8- -0.6 | 34 | 21 | 7 | 6 |
| -0.6- -0.4 | 34 | 21 | 5 | 4 |
| -0.4- -0.2 | 21 | 9 | 8 | 7 |
| -0.2 – 0.0 | 17 | 15 | 4 | 3 |
| 0.0- 0.2 | 12 | 22 | 4 | 2 |
| 0.2-0.4 | 14 | 16 | 1 | 3 |
| 0.4-0.6 | 15 | 24 | 4 | 7 |
| 0.6-0.8 | 19 | 41 | 4 | 13 |
| 0.8-1.0 | 44 | 170 | 11 | 34 |
| 1.0-1.2 | 81 | 538 | 13 | 82 |
| 1.2-1.4 | 6 | 30 | 0 | 2 |

**Figure S1. Schema illustrating the AAC calculations for N, C terminals and the remaining central portion of a protein of length l. *k* varies from 10, 15, 20, 25**
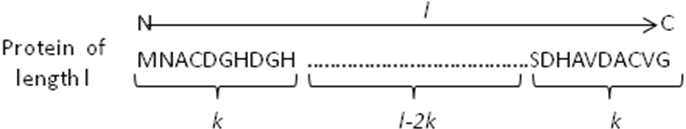
**and 30 residues.**

**Figure S2. The difference in average amino acid composition for virulent and non-virulent proteins**


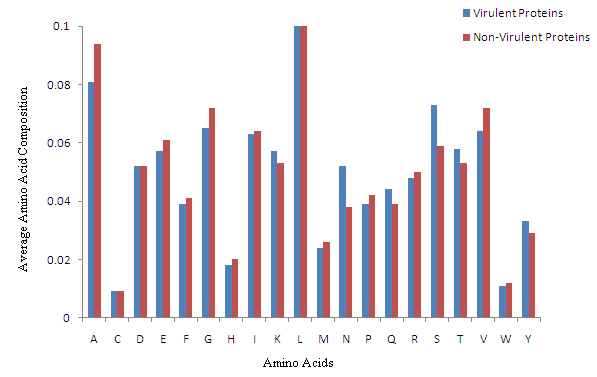


**Figure S3. The number of proteins predicted in different ranges of SVM scores using complete 2055 sequences.**


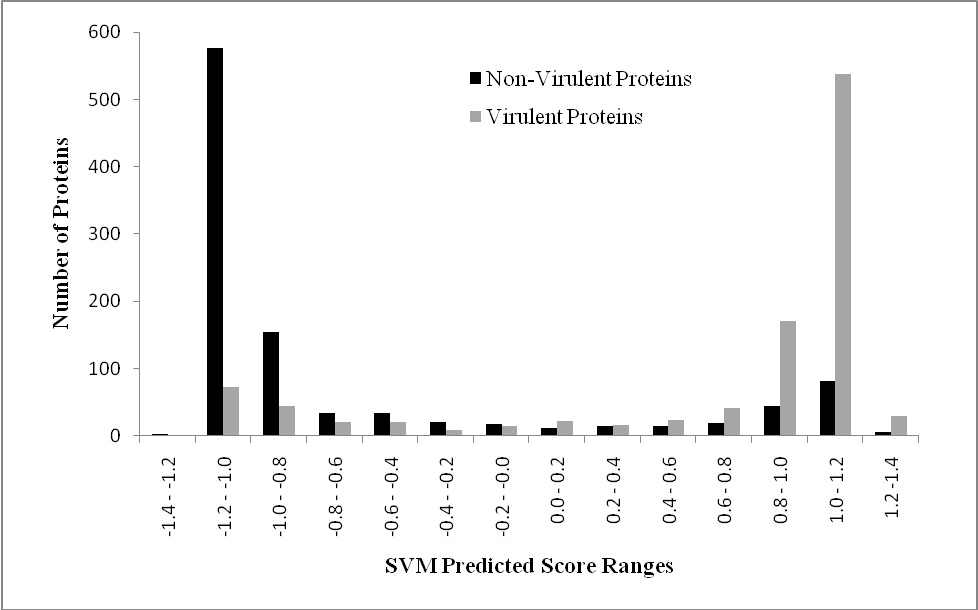

Supplement: Additional file 1 — Six tables (Table S1–S6) giving: effect of compositions derived from different N and C-terminal lengths on the performance of SVM based module (Table S1), the results obtained using traditional and higher order dipeptide composition based SVM modules (Table S2), the performance of PSI-BLAST searches using different iteration values (Table S3), detailed results obtained for Cascade SVM module at different threshold values (Table S4), the parameters of optimized SVMs obtained using different individual features and its combination (Table S5), and the distribution of 2055 proteins and Independent datasets (combined) into different ranges in the scale of -1.4 to 1.4 according to their SVM predicted scores for cascade SVM module (Table S6). The file also contains three figures (Figure S1–S3), giving: Schema illustrating the strategy to calculate AAC of N, C and middle regions of a protein (Figure S1), The difference in average amino acid composition for virulent and non-virulent proteins (Figure S2), The number of proteins predicted in different ranges of SVM scores using complete 2055 sequences (Figure S3). [file 1471-2105-9-62-S1.doc]
